# The effect of nimesulide on skeletal muscle hypertrophy and load progression after 8 weeks of resistance training in wistar rats

**DOI:** 10.1007/s10974-026-09724-3

**Published:** 2026-03-04

**Authors:** Ayron Motta da Fonseca, Ronaldo André Castelo dos Santos de Almeida, Emerson Lopes Olivares, Anderson Luiz Bezerra da Silveira

**Affiliations:** 1 Laboratory of Physiology and Human Performance, Department of Physical Education and Sports, Federal Rural University of Rio de Janeiro, Seropédica, Brazil; 2 Laboratory of Physiology and Cardiovascular Pharmacology, Department of Physiological Sciences, Federal Rural University of Rio de Janeiro, Seropédica, Brazil

**Keywords:** Resistance training, Nimesulide, Hypertrophy, Muscle strength

## Abstract

Resistance training is currently a widely targeted practice due to its ability to induce vital adaptations resulting from increased muscle strength (MS) and hypertrophy. There are reports of the concomitant use of nonsteroidal anti-inflammatory drugs with this practice for pain relief, which arises from the adaptive signaling of training. Based on this, the present study aimed to investigate whether the use of nimesulide concomitant with training could interfere with MS and hypertrophy. The sample consisted of 18 male Wistar rats, aged 91 days, with a body mass of 331 ± 20 g, divided into three groups: Control (CTRL, *n* = 4); Trained (TR, *n* = 7); and Trained and Treated with nimesulide (COMB, *n* = 7). The training protocol followed the ladder-climbing model. Nimesulide was administered orally at a dose of 2.5 mg/kg every day after training. Tissue collection of the Flexor Hallucis Longus (FHL) muscle was performed two days after the last training session. After eight weeks of the training protocol, a significant increase in MS was observed in TR and COMB compared to CTRL (*p* < 0.05). A significant increase in FHL muscle volume was observed in COMB compared to CTRL and TR only when muscle mass was normalized to body mass (*p* < 0.05), whereas no significant differences were detected among groups for absolute FHL mass. Additionally, there were no differences in training load or volume between COMB and TR (*p* ≥ 0.05). Therefore, based on the results, it is concluded that the administration of nimesulide during eight weeks of training promoted a greater hypertrophic response of the FHL muscle in rats.

**Supplementary Information:**

The online version contains supplementary material available at 10.1007/s10974-026-09724-3.

## Introduction

Resistance training is widely recognized as an effective strategy for maintaining health due to its potential to promote hypertrophy and increase muscle strength. (Schoenfeld 2010; Westcott 2012). This training modality involves exercises in which muscle fibers act against an external resistance, typically imposed by specific equipment (Fleck and Kraemer 2014, p. 1). The mechanical stimuli generated by such exercises cause microlesions in the myofibrils, leading to exercise-induced muscle damage (EIMD) and, consequently, delayed-onset muscle soreness (DOMS), which are associated with pro-inflammatory responses (Mizumura and Taguchi 2024; Schoenfeld 2012). These responses promote both morphological and functional adaptations in skeletal muscle tissue (Sartori et al. 2021).

The subsequent inflammatory signaling — characterized by pain, edema, and redness (Wallach et al. 2014) — is mediated by increased prostaglandin production through cyclooxygenase (COX) activity, particularly the PGE₂ pathway, which plays an essential role in muscle regeneration via satellite cell activation (Ho et al. 2017; Mendias et al. 2004; Rodemann and Goldberg 1982).

Given the relationship between prostaglandins and pain, the use of nonsteroidal anti-inflammatory drugs (NSAIDs) to inhibit COX following muscle injury is common. These agents may selectively or non-selectively inhibit COX isoforms — COX-1 (constitutive) and COX-2 (inflammation-related) (Hinz and Brune 2002; Vane et al. 1998). Notably, frequent use of NSAIDs has been reported among athletes, with approximately 50% of participants in the FIFA World Cup reporting usage (Lundberg and Howatson 2018).

COX-2 activity contributes positively to hypertrophic signaling and recovery from muscle atrophy (Bondesen et al. 2006). However, selective COX-2 inhibition may attenuate inflammatory responses, reduce myoblast proliferation, and decrease protein synthesis (Bondesen et al. 2004; Novak et al. 2009; Soltow et al. 2006). Studies have demonstrated that COX-2 inhibition impairs muscle regeneration and hypertrophy in animal models subjected to muscle ablation or injury (Bondesen et al. 2004; Novak et al. 2009; Soltow et al. 2006). In humans, NSAIDs have shown modest effects on musculoskeletal adaptations, although ibuprofen has been reported to reduce myosin expression in the quadriceps (Roberts et al. 2025). Studies indicate that these effects vary according to age and physiological context. In older adults, chronic use of ibuprofen or acetaminophen during resistance training did not compromise gains in muscle mass and strength, despite a reduction in acute protein synthesis (Trappe et al. 2011), nor did it impair muscular and bone adaptations in older women (Duff et al. 2017). In young adults, ibuprofen did not significantly alter hypertrophy or muscle strength, although it reduced delayed-onset muscle soreness (Krentz et al. 2008).

Nimesulide, a selective COX-2 inhibitor, is commonly used in humans to treat for osteoarthritis, low back pain, and postoperative pain (Bianchi and Broggini 2003; Binning 2007; Pohjolainen et al. 2000; Santos et al. 2022). It has demonstrated superior and faster analgesic potential compared to other selective NSAIDs (Bianchi and Broggini 2003; Binning 2007; Santos et al. 2022). However, there is a scarcity of research evaluating its effects on muscle adaptations induced by resistance training.

Therefore, this study aimed to investigate the effects of nimesulide on musculoskeletal responses in a rodent model of resistance training, contributing to the understanding of COX-2 inhibition during hypertrophic adaptation. The null hypothesis (H₀) posits that nimesulide does not alter strength or muscle hypertrophy gains, while the alternative hypothesis (H₁) assumes that the drug negatively influences these adaptations.

## Methods

### Ethical aspects

The study protocol was submitted to and approved by the Ethics Committee of the Federal Rural University of Rio de Janeiro under protocol No. 01/2025. All procedures were conducted in accordance with the ethical principles established by the National Council for the Control of Animal Experimentation (CONCEA) and complied with current legislation.

### Sample

Initially, twenty-one male Wistar Albino rats (Rattus norvegicus), 13 weeks old (91 days) and weighing 331 ± 20 g, were used in this study. An a priori sample size calculation was performed using G*Power software (version 3.1.9.7) for a one-way ANOVA. To comply with the ethical principle of Reduction (3Rs), the calculation predicted that a total of 21 animals (*n* = 7 per group) would be sufficient to detect a large effect size (f = 0.751) with a statistical power of 0.80 and a significance level of α = 0.05.

Animals were randomly assigned to three experimental groups: Trained (TR, *n* = 7), Trained + nimesulide (COMB, *n* = 7), and Control (CTRL, *n* = 7). However, due to unforeseen mortality unrelated to the experimental procedures, three animals from the CTRL group were lost during the protocol. To maintain temporal synchronization and avoid confounding variables associated with a new experimental cohort, these animals were not replaced, resulting in a final sample of *n* = 4 for the CTRL group. Statistical analyses for unbalanced designs were applied to ensure the validity and viability of the results.

### Experimental design

The study was conducted in two stages. The first stage consisted of a seven-day adaptation period to familiarize the animals with the procedures. Animals were randomized in a blinded manner. All groups were habituated to the resistance training ladder and gavage administration to minimize stress-related effects. Animals were housed under controlled conditions (22 °C; 12:12 h light-dark cycle) with ad libitum access to food and water.

**Fig. 1:**
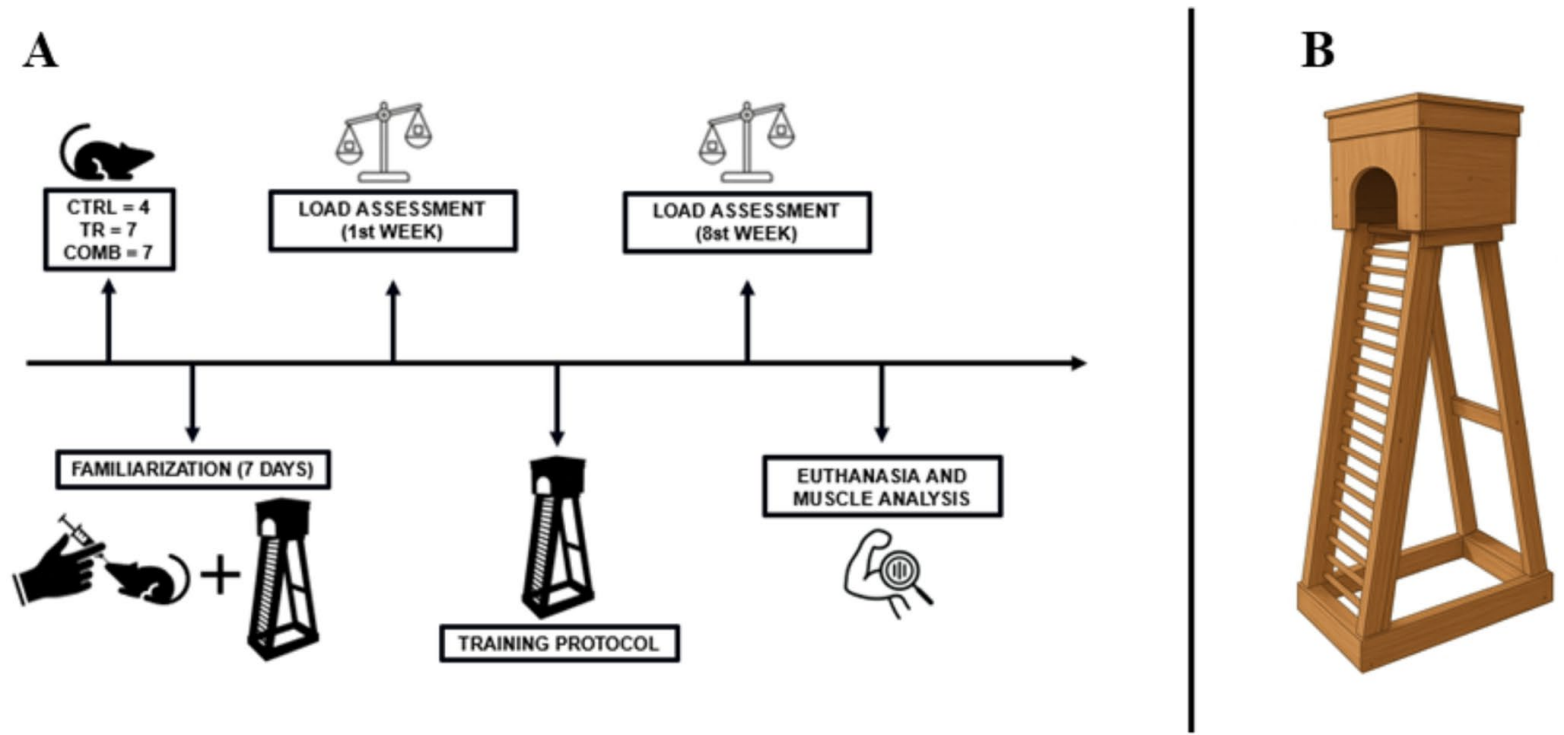
Methodological design and exercise training apparatus. **(A)** Experimental study design. **(B)** Ladder-based resistance training apparatus

The second stage involved pre- and post-training strength assessments in the trained and control groups. The training protocol lasted eight weeks, with one session every three days (totaling 20 sessions) (Fig. 1A).

### Drug preparation and administration

Nimesulide (Cimed^®^, Brazil) was administered at a therapeutic dose of 2.5 mg/kg (50 mg/mL) (Dhir et al. 2007; Saghaei et al. 2012). A 1:20 dilution was prepared by mixing 1 mL of the stock solution with 19 mL of 0.9% saline. The solution was stirred magnetically (INTLAB™) and administered via orogastric gavage after each training session throughout the eight-week period. To ensure dosage accuracy, body weight was monitored weekly and doses were adjusted accordingly. The treated group received the drug, while the control and trained groups received 0.9% saline to control the stress effects associated with gavage.

### Familiarization with the resistance training apparatus

The resistance exercise apparatus consisted of a ladder measuring 110 cm in length, with a housing chamber at the top and an entry hole at the base (Fig. 1B). Prior to performing the maximum load test, the animals underwent a familiarization period with the experimental apparatus. After that, the animals performed three ladder climbs per session over seven consecutive days, carrying tail-attached fabric pouches containing adjustable lead weights for each training session. The pouch featured two straps that were fixed at the proximal portion of the animals’ tails.

### Maximum capacity test

Following Hornberger and Farrar (2004), animals began the test with a load corresponding to 75% of their body weight (BW). Load increments of 30 g (~ 10 ± 2% BW) were added until failure to complete the climb. The maximum load successfully lifted was recorded as the maximum carrying capacity. Tests were performed in the 1 st and 20th sessions.

### Resistance training protocol

Starting at 91 days of age, the TR animals underwent ladder-climbing resistance training three times per week for eight weeks. Each session consisted of 4–9 climbs with progressively increasing loads (50%, 75%, 90%, and 100% of maximum capacity), with 2-minute rest intervals. Subsequent climbs added 30 g until a new maximum was achieved. Thus, the rats’ new maximum load capacity was calculated in each training session. This regimen was repeated every three days for eight weeks, totaling 20 training sessions (Hornberger and Farrar 2004).

### Volume load

Volume load is a quantitative measure of the total work performed in an exercise, muscle group, or resistance training session. It combines volume (number of sets and repetitions) with intensity (weight lifted). It is calculated as the product of the number of sets, the number of repetitions, and the load used (in kilograms or pounds). This variable is used to more comprehensively quantify the training stimulus, prescribe and periodize training to allow progressive adjustments, monitor overload over time as an indicator of progression or fatigue, and compare workload across exercises, sessions, or training cycles (Fleck and Kraemer 2014, p. 6; Krause Neto et al. 2018).

### Euthanasia and post-mortem analysis

Within three days of completing the final session, animals were euthanized by decapitation. The Flexor Hallucis Longus (FHL) muscle was excised, rinsed in 0.9% saline, weighed, and stored at − 80 °C for future histological and biomolecular analyses.

### Statistical analysis

Normality was assessed using the Shapiro–Wilk test. Body mass, maximum absolute load, and maximum normalized load were analyzed using two-way ANOVA (group × time), with repeated measures on the time factor, followed by appropriate post hoc tests. Tukey’s multiple comparisons test was applied for between-group comparisons, while Bonferroni corrections were used for within-group comparisons across time, as appropriate. Volume load across training sessions was analyzed using two-way ANOVA followed by Tukey’s post hoc test. FHL hypertrophy was analyzed using one-way between-groups ANOVA followed by Tukey’s post hoc test. Statistical significance was set at *p* ≤ 0.05. Data are presented as mean ± standard error of the mean (SEM) and were analyzed using GraphPad Prism version 9.0 (GraphPad Software, CA, USA).

## Results

### Body mass

**Fig. 2:**
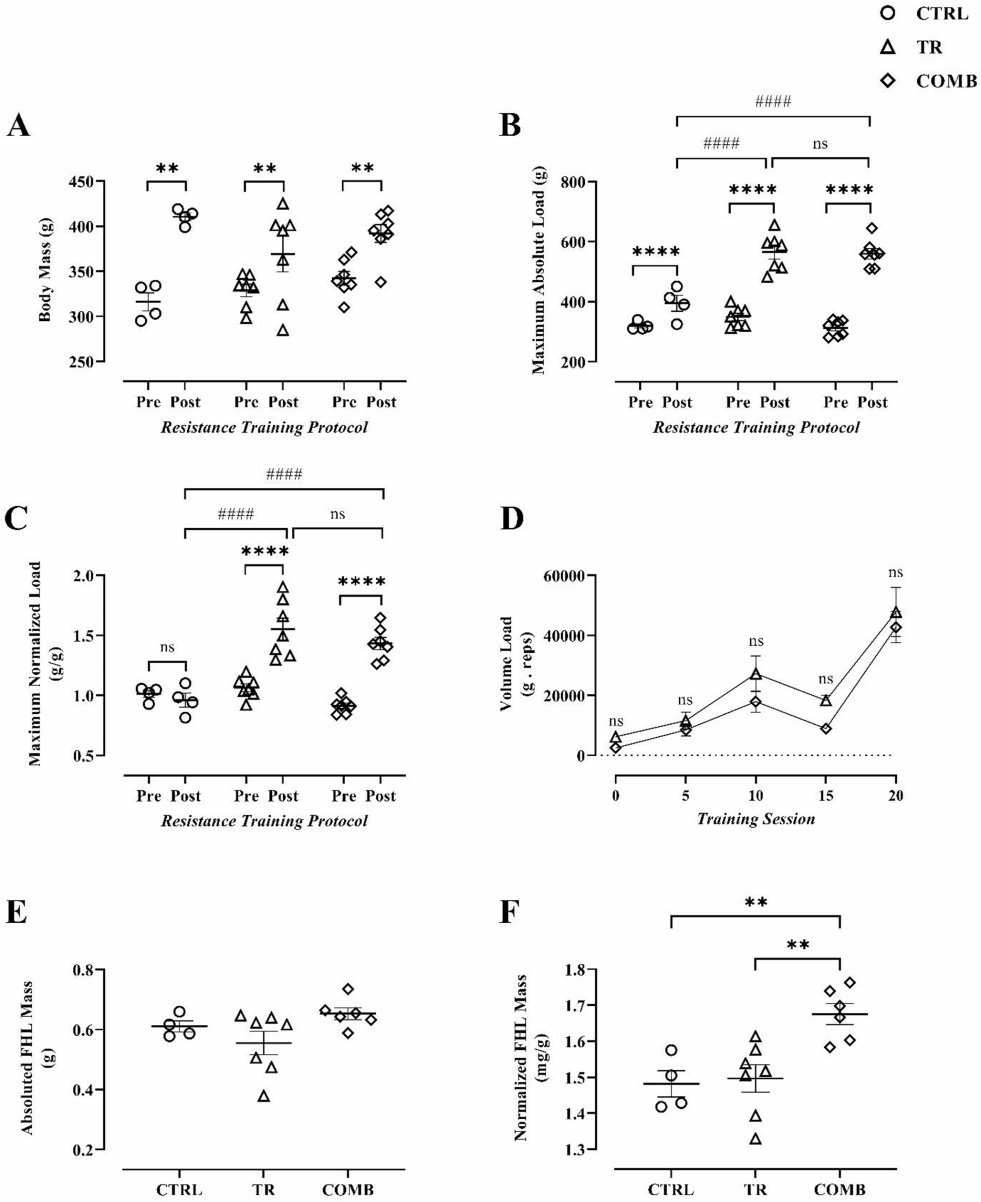
Assessment of muscle strength and hypertrophy to resistance training associated with the use of nimesulide before and after the 8-week of ladder-based resistance training. **(A)** Animals’ body mass; **(B)** Maximum Absolute Load (maximum absolute strength); **(C)** Maximum Normalized Load (maximum normalized strength); (**D)** Volume Load [repetitions (no.) × external load (g)]; **(E)** Absolute FHL Mass; **(F)** Normalized FHL Mass. CTRL: control group; TR: resistance trained group; COMB: resistance trained + nimesulide group; FHL: Flexor Hallucis Longus. **Significant difference within-group (*p* < 0.01). ****Statistical difference within-groups (*p* < 0.0001). ^####^Statistical difference between-groups (*p* < 0.0001). ^ns^non-statistical difference (*p* > 0.05). Data are presented as mean ± standard error of the mean (SEM)

A two-way ANOVA revealed a significant time × group interaction for body mass (*p* < 0.05). Post hoc analysis using the Bonferroni correction demonstrated a significant increase in body mass after eight weeks of training within all groups (CTRL, *p* < 0.0001; TR, *p* = 0.0029; COMB, *p* = 0.0005). Tukey’s post hoc test revealed no significant differences between groups at either time point (Pre: CTRL vs. TR, *p* = 0.7725; CTRL vs. COMB, *p* = 0.3430; TR vs. COMB, *p* = 0.6689; Post: CTRL vs. TR, *p* = 0.0800; CTRL vs. COMB, *p* = 0.5776; TR vs. COMB, *p* = 0.3293) (Fig. 2A).

### Maximum absolute load

For absolute maximum strength, a two-way ANOVA identified a significant time × group interaction (*p* < 0.0001). Subsequent Bonferroni post hoc tests revealed that all groups experienced significant strength gains over the eight-week resistance training protocol (CTRL, *p* = 0.0333; TR, *p* < 0.0001; COMB, *p* < 0.0001). While Tukey’s post hoc test found no intergroup differences at baseline (Pre: CTRL vs. TR, *p* = 0.5077; CTRL vs. COMB, *p* = 0.9662; TR vs. COMB, *p* = 0.2572), post-training results (Post) showed that TR and COMB achieved significantly higher strength levels than CTRL (CTRL vs. TR, *p* < 0.0001; CTRL vs. COMB, *p* < 0.0001), with no disparity between the two trained groups (*p* = 0.9693) (Fig. 2B).

### Maximum normalized load

Regarding the maximum normalized load, the two-way ANOVA indicated a significant interaction between time and group (*p* < 0.0001). Analysis with Bonferroni correction demonstrated that the normalized load increased significantly in the TR and COMB groups (both *p* < 0.0001), while the CTRL group remained unchanged (*p* = 0.9379). Tukey’s post-hoc test confirmed that all groups were statistically indifferent at baseline, showing no significant differences at the beginning of the study (Pre: CTRL vs. TR, *p* = 0.8279; CTRL vs. COMB, *p* = 0.4958; TR vs. COMB, *p* = 0.1226). In contrast, in the post-training assessment (Post), both the TR group and the COMB group showed significantly higher loads than the CTRL group (CTRL vs. TR, *p* < 0.0001; CTRL vs. COMB, *p* < 0.0001), while no significant difference was observed between the two trained groups (*p* > 0.05) (Fig. 2C).

### Volume load

Regarding volume load (Fig. 2D), two-way ANOVA revealed no significant interaction between group and time (*p* > 0.05). However, significant main effects of time (*p* = 0.0002) and group (*p* = 0.0108) were observed. Baseline assessment (session 0) showed no initial differences between groups (TR: 6.200 ± 1.160 vs. COMB: 2.476 ± 469.4; *p* > 0.9999). Although workload was repeatedly assessed to track performance, Tukey’s post hoc test confirmed no significant differences at specific sessions: 5th (TR: 11.558 ± 2.864 vs. COMB: 8.481 ± 2.016), 10th (TR: 27.266 ± 5.887 vs. COMB: 17.898 ± 3.602), 15th (TR: 18.287 ± 1.817 vs. COMB: 8.942 ± 813.3), and 20th (TR: 47.821 ± 8.191 vs. COMB: 42.738 ± 5.220), with *p* > 0.05 for all comparisons.

### Hypertrophy of the Flexor Hallucis Longus (FHL)

FHL muscle hypertrophy was evaluated through absolute mass (Fig. 2E). One-way ANOVA for absolute FHL mass showed no significant differences among groups (*p* = 0.0997). Consistently, Tukey’s post hoc test revealed no significant differences in pairwise comparisons: CTRL vs. TR (*p* = 0.4903), CTRL vs. COMB (*p* = 0.6674), and TR vs. COMB (*p* = 0.0850).

In contrast, when hypertrophy was evaluated using FHL mass normalized to body mass (Fig. 2F), one-way ANOVA revealed a significant difference among groups (*p* = 0.0035). Tukey’s post hoc analysis showed that the combined resistance training and nimesulide group (COMB) exhibited significantly greater normalized FHL mass compared with both CTRL (*p* = 0.0093) and TR (*p* = 0.0058). No significant difference was observed between the CTRL and TR groups (*p* = 0.9580).

## Discussion

The present study primarily aimed to evaluate the possible effects of nimesulide on muscle strength and hypertrophy following eight-week of resistance training.

In this context, significant differences in body mass were observed within all groups, as expected given the experimental duration (Hornberger and Farrar 2004). However, pre-training between groups comparisons revealed no significant differences, confirming sample homogeneity prior to the intervention.

Furthermore, it was observed that the maximal load capacity in response to resistance training was not significantly affected by the continuous administration of nimesulide compared to the trained group that did not receive pharmacological treatment. These results are consistent with human studies, such as that of Paulsen et al. (2010), who reported that NSAID use did not significantly alter strength adaptations to resistance training. Similar findings were reported by Krentz et al. (2008), who demonstrated that ibuprofen administration did not impair gains in muscle strength or hypertrophy during a resistance training program in young adults.

However, these findings contrast with experimental evidence indicating that COX-2 inhibition may attenuate hypertrophic signaling, satellite cell activation, and muscle regeneration, particularly in models of muscle injury or ablation (Bondesen et al. 2004; Novak et al. 2009; Soltow et al. 2006). This discrepancy suggests that the impact of NSAIDs on muscle adaptation is highly dependent on factors such as species, age, training status, injury severity, dosage, and duration of drug exposure.

Regarding the analysis of volume load, defined as the product of the total number of sets performed and the total load lifted (Fleck and Kraemer 2014, p. 6; Krause Neto et al. 2018), the comparison between the TR and COMB groups did not show significant differences. This finding suggests that the analgesic effect of nimesulide did not enhance training tolerance or workload capacity. This observation aligns with previous investigations indicating that NSAID consumption does not significantly modify training volume or perceived effort during resistance exercise (Krentz et al. 2008; Trappe et al. 2011). It is noteworthy that the CTRL group was not included in the comparative analyses of training volume, as it did not perform ladder-based resistance training sets. The CTRL group participated only in the maximal strength assessments before and after the intervention.

With respect to the capacity to promote muscle hypertrophy following eight weeks of intervention, the findings of this study stand in contrast to the conclusions of Hornberger and Farrar (2004), who examined the hypertrophy of the FHL muscle. Nonetheless, the current findings align with other investigations that did not report significant increases in muscle mass in rats subjected to resistance training protocols (Tamaki et al. 1992; Duncan et al. 1998; Bennell et al. 2000). A possible explanation for these results lies in the high degree of muscle damage induced by the training protocol, where the recovery interval between sessions may have been insufficient to allow adequate repair—a prerequisite for hypertrophic adaptations. In this context, neutrophils are likely the first immune cells to infiltrate the injured muscle tissue, particularly in the region of the EIMD (Hyldahl and Hubal 2014). These neutrophils, activated through Ca²⁺-mediated proteolysis (Gissel and Clausen 2001), and act by phagocytosing necrotic myofibers, thereby promoting the muscle regeneration process (Pizza et al. 2005). However, neutrophils can also produce high concentrations of cytolytic and cytotoxic molecules through superoxide anion–dependent mechanisms derived from NADPH oxidase, potentially exacerbating existing tissue damage, as demonstrated in supplementary material 1 (Nguyen and Tidball 2003).

Although no statistically significant differences in muscle hypertrophy were observed between the TR and CTRL groups, the COMB group exhibited greater relative hypertrophy only when FHL mass was normalized to body mass. Importantly, this finding does not indicate a detrimental effect of nimesulide on hypertrophic adaptations, suggesting that the drug did not negatively influence resistance training–induced muscle hypertrophy in the predominantly type II fiber–composed FHL muscle. These results are consistent with those reported by Trappe et al. (2011) and Duff et al. (2017), who demonstrated that chronic NSAID administration did not impair, and in some cases enhanced, muscle and bone adaptations to resistance training in older adults.

It is important to note that cyclooxygenase-2 (COX-2) is directly associated with prostaglandin E₂ synthesis, which in turn is linked to the production of interleukins IL-23 and IL-17. Moreover, neutrophil migration to sites of tissue injury is closely related to IL-23/IL-17 levels (Lemos et al. 2009). Thus, inhibition of prostaglandin synthesis would likely reduce neutrophil migration to the injured area, thereby attenuating the progression of tissue damage. The diagram of this modulation can be seen in the supplementary material (Figure Supplementary 1). Therefore, it is possible to hypothesize that the hypertrophic effect observed in rodents subjected to resistance training and treated with nimesulide in the present study may be attributed to the anti-inflammatory action of nimesulide, a potent selective COX-2 inhibitor, which could promote a signaling cascade that favors muscle hypertrophy. The diagram of this modulation can also be seen in the supplemental material. (Figure Supplementary 2). This mechanistic framework may help explain why selective attenuation of excessive inflammation could create a more favorable microenvironment for muscle regeneration, contrasting with experimental models in which profound COX-2 inhibition severely blunts early regenerative signaling.

Additionally, it is reasonable also to hypothesize that the muscle hypertrophy observed in COMB group in the present study is analogous to the findings of Mackey et al. (2016), who demonstrated that ibuprofen administration in humans, although not COX-selective but capable of inhibiting COX-2 (Orlando et al. 2015), promoted greater activation of the Notch1 signaling pathway and increased satellite cell mobilization.

Taken together, the findings of this study contribute new insights into the pharmacological and physiological mechanisms underlying nimesulide’s effects and provide novel parameters for understanding how resistance training in rodents interacts with NSAID administration.

## Conclusion

Eight weeks of ladder-based resistance training combined with continuous administration of nimesulide (2.5 mg/kg once every three days) induced significant skeletal muscle hypertrophy in Wistar rats. These findings suggest that nimesulide, a selective COX-2 inhibitor, may exert a potential anabolic modulatory effect on training-induced inflammation, thereby promoting hypertrophic adaptations. Further studies are required to elucidate the molecular mechanisms underlying these effects, particularly those related to satellite cell dynamics and protein synthesis signaling, and to explore their translational implications for human resistance training and rehabilitation contexts.

### Study limitations

The relatively small number of animals (*n* = 18) limits the generalizability of the findings and may reduce statistical power for detecting subtle physiological differences. The study primarily focused on functional outcomes (strength and hypertrophy) without quantifying molecular markers related to muscle protein synthesis, inflammatory signaling, or satellite cell activation.

## Supplementary Information

Below is the link to the electronic supplementary material.

Supplementary Material 1 The figure illustrates the signaling cascade triggered in response to a high level of muscle damage resulting from the high overload imposed by the resistance training protocol employed in this study. Excessive skeletal muscle damage leads to a significant increase in the expression of the enzyme COX-2, which is directly and positively associated with the synthesis of prostaglandin E2. This, in turn, stimulates the production of interleukins, especially IL-23 and IL-17. Consequently, neutrophil migration to the regions affected by tissue injury is directly correlated with the levels of these interleukins. However, neutrophils, which usually act in the removal of necrotic myofibers, can also release high concentrations of cytolytic and cytotoxic molecules, through mechanisms dependent on the superoxide anion generated by NADPH oxidase, which can intensify existing tissue damage and promote a necrotic environment. On the left, the mechanism observed in the absence of excessive muscle damage is represented, which favors an environment conducive to hypertrophy. This condition is commonly observed in training programs that include adequate recovery intervals between applied overloads.

Supplementary Material 2 The figure illustrates the pharmacological blocking mechanism exerted by nimesulide on the signaling cascade triggered by excessive muscle damage. Inhibition of high COX-2 concentrations regulates the entire subsequent process to physiologically appropriate levels, consequently reducing the production of cytotoxic cells and fostering an environment conducive to muscle hypertrophy.

## Data Availability

No datasets were generated or analysed during the current study.
