# Supplementary figures and images for: The effect of nimesulide on skeletal muscle hypertrophy and load progression after 8 weeks of resistance training in wistar rats
Source: J Muscle Res Cell Motil. 2026 Mar 4;47(1):6. doi: 10.1007/s10974-026-09724-3 (PMC12960334; doi:10.1007/s10974-026-09724-3)

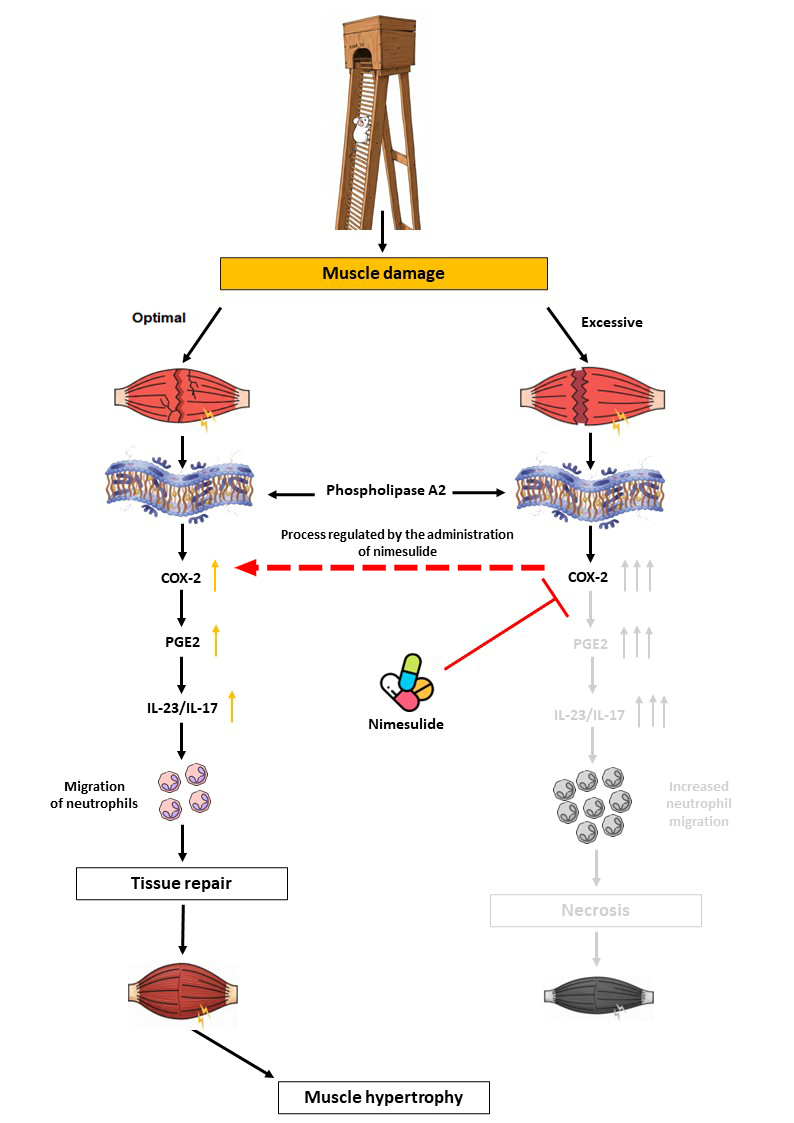

Supplement: Supplementary file 1 — Supplementary Material 1 The figure illustrates the signaling cascade triggered in response to a high level of muscle damage resulting from the high overload imposed by the resistance training protocol employed in this study. Excessive skeletal muscle damage leads to a significant increase in the expression of the enzyme COX-2, which is directly and positively associated with the synthesis of prostaglandin E2. This, in turn, stimulates the production of interleukins, especially IL-23 and IL-17. Consequently, neutrophil migration to the regions affected by tissue injury is directly correlated with the levels of these interleukins. However, neutrophils, which usually act in the removal of necrotic myofibers, can also release high concentrations of cytolytic and cytotoxic molecules, through mechanisms dependent on the superoxide anion generated by NADPH oxidase, which can intensify existing tissue damage and promote a necrotic environment. On the left, the mechanism observed in the absence of excessive muscle damage is represented, which favors an environment conducive to hypertrophy. This condition is commonly observed in training programs that include adequate recovery intervals between applied overloads. [file 10974_2026_9724_MOESM1_ESM.tif]

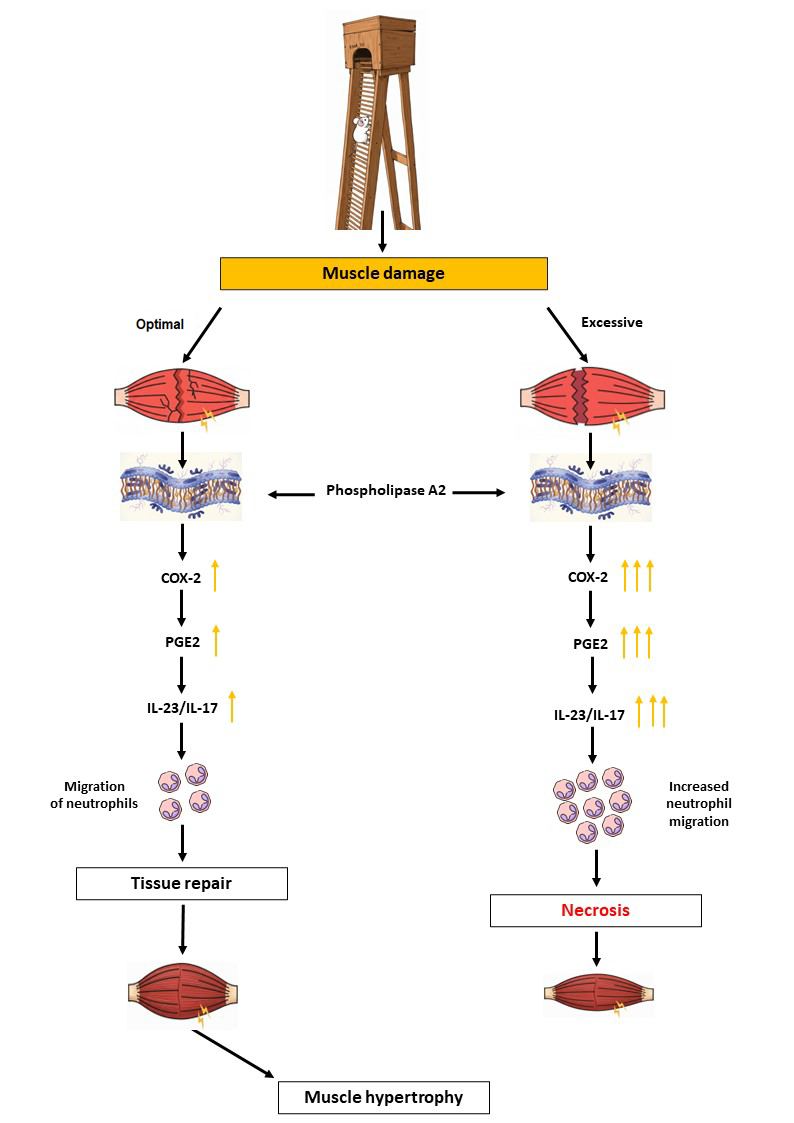

Supplement: Supplementary file 2 — Supplementary Material 2 The figure illustrates the pharmacological blocking mechanism exerted by nimesulide on the signaling cascade triggered by excessive muscle damage. Inhibition of high COX-2 concentrations regulates the entire subsequent process to physiologically appropriate levels, consequently reducing the production of cytotoxic cells and fostering an environment conducive to muscle hypertrophy. [file 10974_2026_9724_MOESM2_ESM.tif]
